# A review of the Masquelet technique in the treatment of lower limb critical-size bone defects

**DOI:** 10.1308/rcsann.2023.0022

**Published:** 2023-06-23

**Authors:** H Ahmed, M Shakshak, A Trompeter

**Affiliations:** St George’s, University of London, UK

**Keywords:** Masquelet, Induced membrane, Critical size, Bone defects, Graft

## Abstract

The need for bone tissue to heal effectively is paramount given its role in the mechanical support of tissues. Bone has a very good natural healing potential in comparison with most other tissue types, largely regenerating to its pre-injury state in the vast majority of cases. Certain factors such as high energy trauma, tumour resection, revision surgery, developmental deformities and infection can lead to the formation of bone defects, where the intrinsic healing potential of bone is diminished owing to bone loss. Various approaches to resolving bone defects exist in current practice, each with their respective benefits and drawbacks. These include bone grafting, free tissue transfer, Ilizarov bone transport and the Masquelet induced membrane technique. This review focuses on evaluating the Masquelet technique, discussing its method and underlying mechanisms, the effectiveness of certain modifications, and its potential future directions.

## Introduction

Bone defects can be classified as partial or segmental. Partial defects involve some contact between the bone ends whereas segmental defects involve no contact between the bone ends.

The definition of what constitutes a “critical-size” bone defect lacks consensus. Suggested definitions include defects that will not heal spontaneously despite surgical stabilisation^1,^^2^ or defects that do not regenerate more than 10% in the lifetime of the animal.^3^ Others argue that it is not a “one size fits all” situation, pointing to the influence that the soft tissue environment and mechanism of injury has over the extent of spontaneous healing of the bone.^4^ Indeed, the popularly accepted rule of >2cm defects being critical arose from animal studies, in which defects were introduced in a controlled manner with minimal trauma to the surrounding soft tissue.^5^ Critical-size defects will progress to non-union if left without intervention and this has a significant impact on the patient’s quality of life.^6^

NHS data show that the total number of diaphyseal lower limb fractures in 2019–2020 was 28,085, with a total of 14,147 bone graft operations in the same period.^7^ This large caseload emphasises the importance of research into the most efficient treatment of bone defects so as to minimise the socioeconomic impact of the disease. Through this review, we have attempted to provide a concise narrative overview of the technique itself as well as current discussions on its optimisation, outcomes and future directions.

## Methods

This paper provides a descriptive, non-systematic review of the current literature on the Masquelet technique and possible directions for future research. A meticulous search through the PubMed^®^ database was performed using the search terms “Masquelet” and “induced membrane”, with peer reviewed articles in English being identified and evaluated. The initial search produced 3,892 results with title and abstract screening carried out by one author. There were no rigid inclusion or exclusion criteria by which to select articles for full-text review. Instead, a subjective assessment was made regarding the relevance of individual papers to a general narrative overview and review of the technique, ultimately resulting in 67 articles being referenced.^1–6,8–68^ Two additional references were sought out, independent of the original search.^7,69^ A further 20 papers were added in response to reviewer comments.^70–89^

## Procedure and osteosynthesis

**Figure 1:**
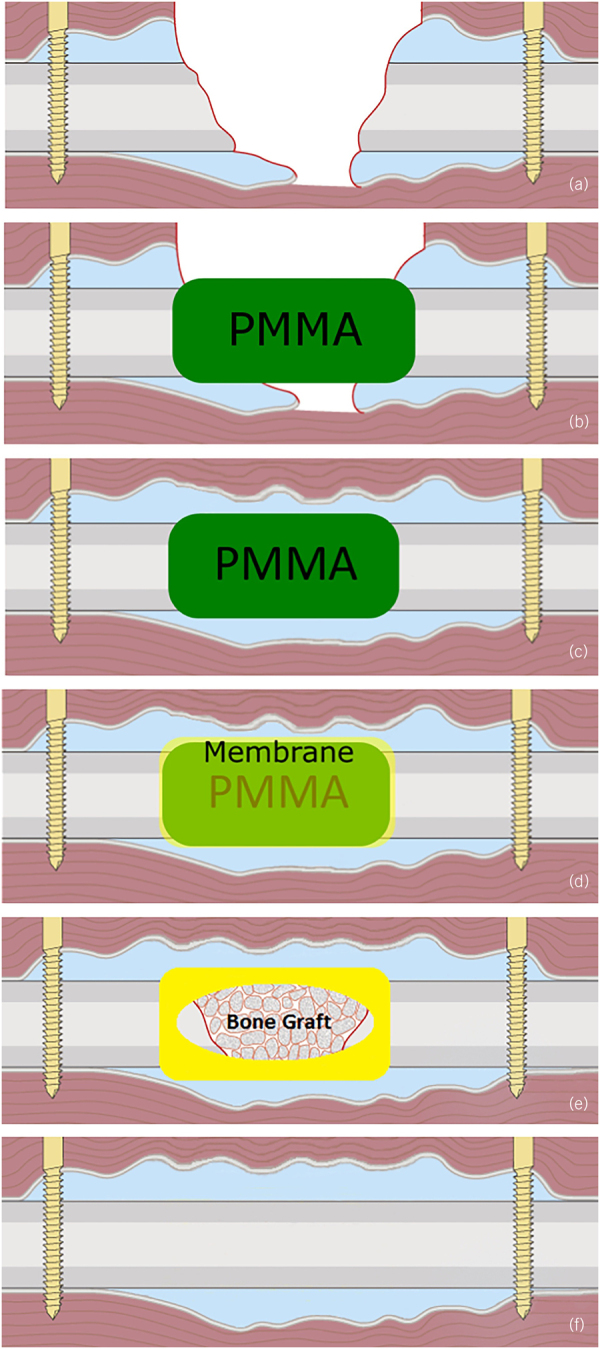
Step-by-step diagram of the Masquelet technique. Stage 1: (a) The defect is exposed, and unhealthy bone and soft tissue are debrided. (b) A polymethylmethacrylate (PMMA) cement spacer is used to fill the void, ensuring that the cement covers the bone ends. (c) Membrane formation: The void is maintained by the PMMA while the surrounding soft tissue heals. (d) Synthesis of the membrane around the spacer. Stage 2: (e) The induced membrane is incised and the spacer is removed. The cavity is then debrided and the bone ends are curetted. The void is filled with appropriate bone graft and the membrane is sutured. (f) Osteointegration of the bone graft. (Reproduced from Tarchala *et al*,^20^ with permission from Wiley.)

The Masquelet technique comprises two stages (Figure 1). Stage 1 involves initial debridement of unhealthy bone and soft tissue while carefully preserving neurovascular structures. A cement spacer is then placed in the bone defect and a bridging fixture is applied. The spacer is typically fashioned and fitted free-hand but this can lead to problems in cases of large defects, where it is difficult to produce a uniformly cylindrical spacer by this technique.

A possible solution to this issue is to split longitudinally a syringe barrel whose diameter roughly matches the diameter of the bone and whose length exceeds the length of the defect by 1–2cm. This simple device is clipped around the proximal and distal fragment ends, and is filled with cement, acting as a mould to help shape the spacer more accurately while also providing some shielding from the thermal energy produced by the curing process.^70^ Once the cement hardens, the syringe barrel can be removed and the spacer is fixed in place with a 2mm Kirschner wire to prevent its migration. The wound is closed and left to heal for a period, during which time a membrane will form around the spacer.

Stage 2 is carried out 4–8 weeks after the initial stage. The membrane is incised longitudinally and the spacer is removed. This reveals a biological chamber bounded by the membrane and the bone ends. The cavity is debrided carefully to avoid disrupting the membrane and the bone ends are curetted to induce bleeding. Autologous bone graft is used to fill the cavity and the membrane is closed. The reconstructed segment is then stabilised adequately during the patient’s recovery.^8^

Stability throughout the procedure is achieved via external fixation or plate fixation, or with an intramedullary nail. Temporary external fixators are recommended in the case of chronic infection.^8^ A study published in 2019 found that intramedullary nail use results in decreased time to union and faster weightbearing (particularly in the femur) compared with plate fixation.^9^ This could be due to the load-sharing-in-axis construct, which produces circumferential stress that promotes the formation of a cortex.

## The induced membrane

The cement spacer implanted in stage 1 is identified as a foreign body, provoking an immune response that leads to the synthesis of a fibrous membrane.^10^ The membrane is composed of a fibroblast/collagen matrix with an inner synovial-like epithelium and a vascularised outer layer as well as being rich in cells like mesenchymal stem cells (MSCs), leucocytes and osteoclasts.^71^ The membrane’s osteogenic potential was discovered serendipitously by Masquelet. While his contemporaries excised the membrane, Masquelet chose to spare it to avoid excessive bleeding. He later deduced that this preservation of the membrane was the reason that his grafting procedures were successful while others experienced graft resorption.^11^

The compartmentalisation of the interfragmentary area by the membrane enhances regeneration by controlling the humoral and cellular environment of the defect. This may diminish activation of osteoclasts by cytokines such as TNF, IL-6 and IL-1, reducing bone graft resorption.^12–14^ Moreover, the membrane prevents ingrowth of fibrous tissue, which is a common cause of fracture non-union.^15,72^

In addition to this barrier effect, the membrane has been described as “pseudosynovial”, secreting osteoinductive factors such as BMP-2, and growth factors such as VEGF and TGF-β1 into the defect. These help revascularise the bone graft and promote bone regeneration.^10^

The osteogenic abilities of the membrane are believed to peak and subsequently diminish. Expression of VEGF drastically decreases after one month^16^ while the expression of BMP-2 peaks at 4–6 weeks.^10,17^ These data indicate that the ideal time to perform the second stage is between four and six weeks after the first stage. However, owing to the variability of injuries in the clinical setting, the timing cannot always be predicted as wound healing is considered a prerequisite before advancing to the second stage.^18^ A compromised soft tissue environment might also delay membrane formation, with the inability to advance until formation is complete.^8^

The clinically accepted consensus is that 4–8 weeks is the ideal time to wait between the two stages of the procedure. On the other hand, the evidence base behind this faces scrutiny as there are examples where the technique has been successful after a much longer waiting period.^73,74^

A study analysing membranes aged between 1 and 16 weeks found histological differences as the membrane ages, including increased vascularisation and fibrosis.^71^ Membranes at all timepoints had MSCs present, indicating osteogenic potential, but the study did not quantify the content of MSCs in each membrane to assess whether that number diminishes with time. It was, however, noted that while all membranes had the ability to stimulate MSC proliferation in culture, this was most marked in the 29–49-day-old membranes, corroborating the idea that membrane osteogenic activity peaks around 4–8 weeks. Concentrations of osteogenic molecules such as carboxy-terminal peptide of collagen type I (CICP), osteocalcin and osteopontin were found to be comparable in all the membranes.

Delaying the second surgery by 6 months has been found to not have any significant impact on the speed of bone healing, indicating that the osteogenic properties of the membrane are retained beyond the recommended 4–8 weeks.^73^ In addition, this delay has the benefit of allowing time for soft tissue healing, infection control and recovery of joint motion.

Tarchala *et al*’s rabbit study compared the biological membrane with a synthetic polytetrafluoroethylene (PTFE) membrane.^15^ They observed no significant difference between the two membranes in ratio of bone volume to tissue volume. However, this study was limited by the small sample size of only five rabbits, and the fact that the micro computed tomography analysis was unable to differentiate between newly formed bone tissue and allograft. Nevertheless, this highlights the continued uncertainty over the precise molecular mechanisms that underpin the Masquelet technique.

## The cement spacer and antibiotics

The cement spacer introduced in the first stage has both a physical role in preventing soft tissue collapse into the bone defect and a biological role in the induction of the membrane.^19^ The type of cement typically used in the Masquelet technique is polymethylmethacrylate (PMMA), which is well established in orthopaedic surgery, having been introduced in 1950.^69^ It can be moulded to fit the defect, before hardening into a stable structure that is able to bear weight.^17^ Disadvantages of PMMA include potential bone necrosis and soft tissue damage from the exothermic reaction it produces when setting as well as a systemic allergy risk.^20^

A possible replacement for PMMA could be a calcium compound spacer. Rat studies show that calcium sulphate produces a histologically similar membrane to PMMA, releasing comparable levels of osteoinductive molecules and growth factors.^17^ It is also biodegradable, obviating the need for its removal in a second stage operation. This is beneficial as piecemeal removal of the PMMA spacer with an osteotome and hammer can weaken the bone.^21^

Calcium sulphate spacers show partial resorption by four weeks and are completely resorbed by eight weeks.^17^ Resorption occurs from the periphery to the core, leaving behind an osteogenic calcium phosphate lattice with granules that are 80μm in diameter.^75,76^

Microparticles released by calcium phosphate on resorption may be phagocytosed and can result in an inflammatory reaction.^77^ During this reaction, the release of inflammatory cytokines is found to promote osteogenesis, for example by the release of TNF, which recruits MSCs.^78^ Other in vitro studies have suggested that microparticle release from calcium phosphate can induce inhibition and damage to surrounding cells, which may affect osteogenesis.^79^ Conversely, subcutaneous and intramuscular injection of a calcium phosphate and polymer solution showed no inflammatory reaction or ectopic bone formation over a period of 1–78 weeks.^75^

Infection is the major complication of the Masquelet technique and is managed via the initial tissue debridement as well as antibiotic elution by the spacer.^22^ Masquelet worries, however, that using antibiotic eluting spacers might mask inadequate debridement. In order to minimise this risk, he recommends using a spacer without antibiotics, alongside oral antibiotic prophylaxis limited to seven days.^23^ The appropriate middle ground therefore appears to be radical debridement in the first stage (with some authors suggesting osteomyelitis be surgically treated as a malignancy) coupled with antibiotic eluting spacers in the second stage.^22,24^

The composition and preparation of the spacer can have an effect on the elution of antibiotics. One of the benefits of calcium sulphate over PMMA is its ability to release the entire antibiotic load as it undergoes complete resorption. Additionally, its curing process is not strongly exothermic, allowing it to deliver heat sensitive antibiotics.^25^ Nevertheless, it should be noted that with the exception of gentamicin, aminoglycosides are largely unaffected by the heat released from PMMA curing. When heated to 83°C, gentamicin undergoes a 25% reduction in concentration but this has minimal effect on its performance in a disc assay. Tobramycin has been suggested as a more appropriate antibiotic to associate with PMMA owing to it having a comparable spectrum of activity with gentamicin while outperforming it in heat stability.^80^

Scanning electron micrography shows that antibiotic molecules become encased in the PMMA matrix as it hardens and only antibiotic molecules close to the surface are readily released. The elution of antibiotics from PMMA follows a biphasic profile, with this initial burst of surface antibiotic followed by a sustained release at subinhibitory concentrations. This may promote antibiotic resistance. Moreover, as it is not resorbed, the PMMA spacer might act as a nidus for bacterial biofilm infection.^25^

Different combinations of antibiotics and cement types can affect the properties of the induced membrane that subsequently forms. Palacos^®^ cement (Heraeus, Wehrheim, Germany) infused with gentamicin led to a 40% increase in membrane thickness between two and six weeks.^26^ Conversely, the combination of Copal^®^ cement (Heraeus) with gentamicin and clindamycin, or Copal^®^ spacem cement with no antibiotic, resulted respectively in a 52% and 59% reduction in thickness over the same period. As well as affecting membrane thickness, the fraction of elastic fibres that comprise the developing membrane is also altered depending on the combination of cement and antibiotic used, but this difference diminishes as the six-week mark approaches.

Rathbone *et al* demonstrated that antibiotics can also have a differential effect on the number and viability of osteogenic cells.^27^ Rifampicin, tetracyclines and ciprofloxacin were shown to decrease cell number as well as alkaline phosphatase levels (used as a marker for osteogenic activity) by 75% at a dose of 100μg/ml. Tobramycin, vancomycin and amikacin were found to be the least decremental to both cell number and osteogenic activity.

The effect of these findings on the success of the Masquelet technique in clinical practice remains to be explored.

## The bone graft

### Ideal source in the Masquelet technique

Morselised cancellous autologous bone graft is the gold standard graft material to fill the defect in the second stage.^8,28,29^ Traditionally, the preferred site of harvest is the iliac crest (IC) owing to ease of access, good graft volume, and an abundance of progenitor cells and growth factors.^30,31^ More recently, reamer/irrigator/aspirator (RIA; DePuy Synthes, Leeds, UK) derived bone graft from the femoral or, less commonly, the tibial canal has gained popularity.^8^

The RIA is a tool that provides continuous irrigation and suction during the reaming of long bones. This decreases the risk of thermal necrosis and venous fat embolism that can occur as a result of the heat and pressure generated by the reaming process.^81^ The irrigant can then be passed through a filtration system allowing the reaming debris to be captured and used as graft material.^32^

### Histological comparisons

Sagi *et al* observed a higher expression of BMP-2 and BMP-4 in RIA bone graft (RIABG) compared with iliac crest bone graft (ICBG).^32^ Meanwhile, ICBG showed higher expression of HIF-1α and VEGF, which are involved in angiogenic pathways, as well as Runx2, which is the master gene regulator of osteoblastogenesis.^32,33^

MSCs in RIA patients may also have a greater osteogenic and chondrogenic differentiation potential compared with IC MSCs, exhibiting higher alkaline phosphatase activity and increased calcification of the extracellular matrix. Toosi *et al* demonstrated that they also exhibited higher expression of COLII and COLX.^34^ However, the small sample size of only three men per tissue type is worth noting when considering the generalisability of these findings.

### Clinical comparisons

In a 2019 study comparing RIABG and ICBG in patients with bone defects of ≤2cm, the rate of union was found to be comparable.^30^ Nevertheless, both the operative time and mean time to union were significantly shorter in the RIA group. Operative time was reduced by 60 minutes on average and the mean time to union was reduced by 1.8 months on average when using the RIA technique. Additionally, a larger randomised prospective trial found no statistically significant difference in union rate or mean time to union between RIA and IC cohorts.^36^

RIABG collected from the femur is associated with a lower incidence of donor site morbidity than for IC procedures. Calori *et al* observed greater postoperative pain in patients who had IC procedures compared with the RIA technique,^37^ a finding that is echoed by other studies.^35,36,38^ Donor site infection and sensory disturbances are also complications that have been observed in ICBG patients.^35,37^ In their meta-analysis, Morelli *et al* found the overall complication rate for RIA procedures to be 6% compared with 19.37% for ICBG.^39^

Complications with the RIA procedure tend to be related to technique. Incorrect trajectory of the reamer can lead to perforation through the femoral cortex that may result in prolonged postoperative pain or may cause damage to the femoral neck requiring prophylactic fixation.^38^ Furthermore, advancing too rapidly down the canal may cause the reamer head to become stuck.^40^ Larger reamer heads harvest greater volumes of graft but may compromise torsional strength if more than the inner 2mm of cortical bone is resected.^41^

When treating defects in the lower limb, harvesting from the ipsilateral femur can help minimise immobility in the patient. If the defect is in the femur itself, that same femur can be considered for harvest with the knowledge that the yield of graft may be lower.^82^

RIA graft is typically harvested from the femoral canal, with the tibial canal also a viable harvest site. RIA harvest from the tibia is discouraged by some owing to the risk of posterior cortical thinning, and the fact that it is more difficult to approach because of the eccentric starting position and inflexibility of the equipment.^82–84^ One study comparing the second iteration of the RIA system in the tibia and the femur of cadaveric specimens found no significant difference between the graft volumes obtained after three passes, but it did find a significant increase in perforations of the tibia (*p*=0.03).^85^

These various studies suggest that RIABG is at least as effective as ICBG, displaying comparable osteogenic properties and outcomes. The shorter operative time and larger volumes of graft harvested in the RIA technique may also offset the higher cost of the apparatus, potentially making the RIA option more cost effective in certain cases.^36^ Taken together with the lower donor site morbidity observed in RIA patients, these findings produce a strong case in favour of RIABG over ICBG.

Some researchers are more hesitant with the RIA technique, citing concerns about blood loss due to the suction applied to the intramedullary canal. A study of 108 patients found the mean estimated blood loss in the RIA group to be over double the blood loss seen in the IC group (*p*<0.001).^42^ Additionally, the likelihood of requiring a transfusion was 5.32 times greater after RIABG harvest compared with an ICBG harvest, raising concerns about transfusion related morbidity.

Further randomised controlled trials comparing the use of RIABG and ICBG in the context of the Masquelet technique are required to definitively assess which is the superior source of graft.

### Graft volume and expanders

A prominent disadvantage of the Masquelet technique compared with Ilizarov bone transport is the need for large volumes of bone graft to appropriately fill the defects. This can be alleviated by using RIABG, which yields greater volumes than ICBG, particularly in children.^40,41^ In the case of massive bone defects, however, these volumes remain insufficient, meaning graft volume expanders are necessary.

Allograft is the usual choice for augmenting graft volume, being readily available with no donor site morbidity. Drawbacks include the risk of transmitting infections like hepatitis C and HIV^43,44^ as well as the possibility of rejection.^45^ These risks can be mitigated by processing the graft through freezing or irradiation although this will remove the graft’s osteogenic potential, and may reduce its osteoinductive properties and mechanical strength.^46^ The osteoconductive properties of allograft matrix are spared, and are useful in the regeneration of bone.

Demineralised bone matrix is allograft that has been processed with acid to decalcify it while retaining the trabecular bone structure, which acts as an osteoconductive scaffold.^46^ Immunogenic rejection is precluded by the removal of surface antigens during demineralisation.^47^ Demineralised bone matrix may exhibit increased osteoinductive properties compared with mineralised allograft as the removal of the mineral phase increases the bioavailability of the underlying osteoinductive factors and collagen.^46^

Synthetic ceramic-based materials such as hydroxyapatite and tricalcium phosphate are also popular graft expanders, their advantages being ample availability and ease of storage, alongside a lack of immunogenicity. However, they generally do not have intrinsic osteoinductive or osteogenic potential, providing only an osteoconductive scaffold.^48^ Nevertheless, the differing resorptive and mechanical properties of tricalcium phosphate and hydroxyapatite can be utilised to produce graft that is tailored to the specific needs of a defect.^49,50^

Further research is required to elucidate the ideal ratio of autograft to graft expander. Current recommendations include 70% autograft to 30% expander,^8^ and tricalcium phosphate content in the graft not exceeding 50%.^48^

A small trial published in 2017 investigated an interesting approach to the problem of insufficient autograft.^51^ The authors noted that the central core of a tightly packed bone graft is too far away from the induced membrane to become vascularised in the early stages of consolidation and is thus resorbed. By replacing this central core with an absorbable gelatine sponge and circumferentially grafting bone around it, the volume of graft required to fill the defect is reduced by 21.4% on average. Moreover, this technique removes the risks of infection and failure of fixatives that are associated with central graft necrosis. The method delivered similar clinical outcomes to previous trials that followed the traditional Masquelet technique. As the study only involved 21 patients with no control group, larger scale randomised controlled trials of this technique may identify it as a simple solution to a pertinent issue in orthopaedic surgery.

## Outcomes and complications

Mi *et al* reviewed cases from 1985 to 2018 totalling 680 defects in 677 patients.^52^ The union rate after the second stage was 88.82%, rising to 92.35% when including 24 cases that achieved union after supplementary procedures. The mean time to union was 245 days after stage 2. The total complication rate was 26.03%, with non-union and deep infection being the most common complications (at 8.97% and 8.09% respectively). The tibia was the site of the most complications but was also represented disproportionately among the cases (402/680), which may have influenced this.

An earlier meta-analysis by Morelli *et al* included 427 patients and found the union rate after stage 2 to be 82%.^39^ This rose to 89.7% when including 33 patients who achieved union after receiving additional union procedures, including repetition of the first step, addition of a fibular graft, or switching to bone transport or other union techniques. The union time ranged from 6 to 211 weeks after stage 2. The rate of complications was 49.6%. This high rate was a result of a wide net being cast, with instances of donor site morbidity and stiffness/decreased range of motion included among complications. Other less prominent complications included fixation material failure, misalignment, length discrepancy and re-fracture.

A major rare complication is bone graft resorption, with three reported cases by Accadbled *et al* cited frequently in the literature.^53^ Graft resorption complications are also reported in other case series^54,55^ but the precise cause remains a source of confusion.

The second stage of the Masquelet technique is recommended to occur 4–8 weeks after the first stage.^8^ In these cases of graft resorption, stage 2 was delayed by up to seven months to allow the completion of adjuvant chemotherapy. As described earlier, histological studies suggest a downward trend in the osteoinductive properties of the membrane as it ages past 4–6 weeks. A suboptimal membrane might therefore have contributed to the graft resorption.

Inadequate osteosynthesis may be implicated in this complication. A 2017 case series reports a patient with congenital fibular pseudarthrosis who had graft resorption after stage 2 of the technique.^56^ A second attempt with a more stable fixation resulted in successful union after ten months, leading the authors to conclude that the stability of bone fixation is the key to success with the Masquelet technique. Finding a solution to this complication would be a step towards increasing confidence in the success rate of the Masquelet technique and reducing uncertainty.

## Conclusions

The Masquelet technique has been shown to be an effective treatment of critical-size defects. Its simplicity is a major boon; it does not require the complex equipment or plastic surgery expertise necessary in Ilizarov bone transport or vascularised bone grafting.^57^ The major advantage over Ilizarov bone transport is that time to union is independent of length of defect, making patient compliance less of an issue even with defects that span up to 25cm.^57,58^ These advantages over its counterparts make it a widely accessible therapy, particularly useful in developing countries.^59^

Where the Masquelet technique falls down against bone transport is its need for two surgical procedures (which may be unsuitable for older patients) while the Ilizarov technique is less invasive and does not require wound dissection. In cases where there is insufficient soft tissue coverage, the Masquelet technique requires plastic surgery input to reconstruct the soft tissue whereas the Ilizarov technique is able to effect distraction histogenesis. Additionally, the Masquelet technique has poor resistance to torsional and shear forces, and needs to be protected by internal or external fixation.^57^

### Future directions

Several options for future directions have been gleaned in the process of writing this review. The timing of the second stage requires further investigation. While optimal membrane activity in regard to growth factor and cytokine release has been narrowed down to between four and eight weeks after the first stage, it would be useful to understand how this correlates with the clinical success of the technique, considering treatment can still be successful even eight years after this period.^60^

A promising method of optimising the Masquelet technique lies in the “diamond concept” of fracture healing.^61^ It suggests that mechanical stability of the fracture site is as important as the combination of osteogenic, osteoinductive and osteoconductive properties of the graft. A carefully thought-out mechanism of osteosynthesis may facilitate the conversion of woven bone to lamellar bone in accordance with Wolff’s law, which might lower the risk of re-fracture.^62^

Advances in materials such as resorbable spacers and synthetic membranes might pave the way to a one-stage procedure, removing the drawback of multiple surgeries and perhaps even the need for bone graft. This could be achieved with degradable spacers like calcium sulphate that induce a similar membrane to PMMA.^63^ Alternative routes include the research of Tarchala *et al* into PTFE membranes^15^ or the use of tissue engineering to produce biomimetic membranes.^64^

A search of the literature found few studies assessing a one-stage Masquelet technique. Verboket *et al* used rat models with femoral defects, comparing the use of human decellularised dermis as a pre-formed membrane with the standard induced membrane, alongside an unenveloped control group.^86^ The structure of human decellularised dermis resembles that of the induced membrane, with high collagen content and tensile strength, and fibrous layers aligned in parallel. After eight weeks, micro computed tomography analysis showed that the best ossification was in the dermis group. Biomechanically, the healed bone in the dermis group was able to withstand similar forces to that in the induced membrane group when compared with their respective contralateral limbs.

Fenelon *et al* compared the use of human amniotic membrane in a one-stage procedure with the classic two-stage Masquelet procedure.^87^ The bioactive nature and natural barrier function of human amniotic membrane in utero makes it attractive as an induced membrane substitute compared with acellular and synthetic options.^88^ Fenelon *et al* found no difference between the results of the two-stage and one-stage techniques.^87^ However, the results are somewhat confounded by their use of a calcium phosphate spacer loaded with BMP-2 to fill the intermembrane space, instead of cancellous autograft (which is understandable given the nature of the small animal model).

While regeneration occurred in all groups bar the empty defect group, the results of the membrane groups were not found to be superior to those of the control group (which had the spacer alone and no membrane).^87^ One cannot therefore be certain whether the membranes had any effect on bone regeneration in the defect as the results are confounded by the use of a spacer that is able to facilitate equivalent regeneration alone, without membrane support. On the other hand, the study did provide evidence that a critical-size bone defect can be regenerated with just a calcium phosphate spacer loaded with BMP-2 in a one-stage procedure.

A recent review from the EU’s Horizon 2020 Smart Bone Regeneration research project highlighted some technical advances in the treatment of critical-size defects that could eventually lead to a one-stage Masquelet technique.^89^ These include the technique of three-dimensional printing of ceramic resorbable calcium sulphate spacers as well as the use of electrospinning to produce synthetic polymer membranes such as polycaprolactone, polylactic acid and polyglycolic acid. The fibrous structure of these synthetic membranes resembles collagen, and the thickness and tensile strength matches that of periosteum. The use of these materials in a one-stage Masquelet procedure remains to be explored.

While the benefits and drawbacks of the various treatment options for critical-size defects have been delineated at length in the literature, prospective comparative trials between these and the Masquelet technique are still necessary to facilitate direct comparisons. The findings of these trials, along with a more robust definition of critical size, would enable a more algorithmic approach to the treatment of bone defects.

## References

[1] Keating JF, Simpson AH, Robinson CM. The management of fractures with bone loss. *J Bone Joint Surg Br* 2005; **87**: 142–150. 15736731 10.1302/0301-620x.87b2.15874

[2] Schmitz JP, Hollinger JO. The critical size defect as an experimental model for craniomandibulofacial nonunions. *Clin Orthop Relat Res* 1986; **205**: 299–308. 3084153

[3] Pneumaticos SG, Triantafyllopoulos GK, Basdra EK, Papavassiliou AG. Segmental bone defects: from cellular and molecular pathways to the development of novel biological treatments. *J Cell Mol Med* 2010; **14**: 2561–2569. 20345845 10.1111/j.1582-4934.2010.01062.x PMC4373476

[4] Schemitsch EH. Size matters: defining critical in bone defect size! *J Orthop Trauma* 2017; **31(Suppl 5)**: S20–S22. 10.1097/BOT.0000000000000978 28938386

[5] Schmidmaier G, Capanna R, Wildemann B *et al.* Bone morphogenetic proteins in critical-size bone defects: what are the options? *Injury* 2009; **40(Suppl 3)**: S39–S43. 20082789 10.1016/S0020-1383(09)70010-5

[6] Babhulkar S, Pande K, Babhulkar S. Nonunion of the diaphysis of long bones. *Clin Orthop Relat Res* 2005; **431**: 50–56. 10.1097/01.blo.0000152369.99312.c5 15685055

[7] NHS Digital. Hospital admitted patient care activity 2019–20. https://digital.nhs.uk/data-and-information/publications/statistical/hospital-admitted-patient-care-activity/2019-20 (cited April 2023).

[8] Masquelet A, Kanakaris NK, Obert L *et al.* Bone repair using the Masquelet technique. *J Bone Joint Surg Am* 2019; **101**: 1,024–1,036. 31169581 10.2106/JBJS.18.00842

[9] Morwood MP, Streufert BD, Bauer A *et al.* Intramedullary nails yield superior results compared with plate fixation when using the Masquelet technique in the femur and tibia. *J Orthop Trauma* 2019; **33**: 547–552. 31403558 10.1097/BOT.0000000000001579

[10] Pelissier P, Masquelet AC, Bareille R *et al.* Induced membranes secrete growth factors including vascular and osteoinductive factors and could stimulate bone regeneration. *J Orthop Res* 2004; **22**: 73–79. 14656662 10.1016/S0736-0266(03)00165-7

[11] Maîtrise Orthopédique. Alain-Charles Masquelet. https://web.archive.org/web/20221228013731/https://mo-journal.com/interviews/alain-charles-masquelet-221 (cited April 2023).

[12] Kim JH, Jin HM, Kim K *et al.* The mechanism of osteoclast differentiation induced by IL-1. *J Immunol* 2009; **183**: 1862–1870. 19587010 10.4049/jimmunol.0803007

[13] Klaue K, Knothe U, Anton C *et al.* Bone regeneration in long-bone defects: tissue compartmentalisation? In vivo study on bone defects in sheep. *Injury* 2009; **40(Suppl 4)**: S95–S102. 19895960 10.1016/j.injury.2009.10.043

[14] Yokota K, Sato K, Miyazaki T *et al.* Combination of tumor necrosis factor α and interleukin-6 induces mouse osteoclast-like cells with bone resorption activity both in vitro and in vivo. *Arthritis Rheumatol* 2014; **66**: 121–129. 24431283 10.1002/art.38218

[15] Tarchala M, Engel V, Barralet J, Harvey EJ. A pilot study: alternative biomaterials in critical sized bone defect treatment. *Injury* 2018; **49**: 523–531. 29153382 10.1016/j.injury.2017.11.007

[16] Aho OM, Lehenkari P, Ristiniemi J *et al.* The mechanism of action of induced membranes in bone repair. *J Bone Joint Surg Am* 2013; **95**: 597–604. 23553294 10.2106/JBJS.L.00310

[17] Ma YF, Jiang N, Zhang X *et al.* Calcium sulfate induced versus PMMA-induced membrane in a critical-sized femoral defect in a rat model. *Sci Rep* 2018; **8**: 637. 29330453 10.1038/s41598-017-17430-x PMC5766563

[18] Obremskey W, Molina C, Collinge C *et al.* Current practice in the management of open fractures among orthopaedic trauma surgeons. Part B: Management of segmental long bone defects. A survey of Orthopaedic Trauma Association members. *J Orthop Trauma* 2014; **28**: e203–e207. 26057886 10.1097/BOT.0000000000000034

[19] Henkel J, Woodruff MA, Epari DR *et al.* Bone regeneration based on tissue engineering conceptions – a 21st century perspective. *Bone Res* 2013; **1**: 216–248. 26273505 10.4248/BR201303002 PMC4472104

[20] Tarchala M, Harvey EJ, Barralet J. Biomaterial-stabilized soft tissue healing for healing of critical-sized bone defects: the Masquelet technique. *Adv Healthc Mater* 2016; **5**: 630–640. 26855349 10.1002/adhm.201500793

[21] Masquelet AC. Induced membrane technique: pearls and pitfalls. *J Orthop Trauma* 2017; **31(Suppl 5)**: S36–S38. 10.1097/BOT.0000000000000979 28938390

[22] Han W, Shen J, Wu H *et al.* Induced membrane technique: advances in the management of bone defects. *Int J Surg* 2017; **42**: 110–116. 28478316 10.1016/j.ijsu.2017.04.064

[23] Apard T, Bigorre N, Cronier P *et al.* Two-stage reconstruction of post-traumatic segmental tibia bone loss with nailing. *Orthop Traumatol Surg Res* 2010; **96**: 549–553. 20605548 10.1016/j.otsr.2010.02.010

[24] Sanders J, Mauffrey C. Long bone osteomyelitis in adults: fundamental concepts and current techniques. *Orthopedics* 2013; **36**: 368–375. 23672894 10.3928/01477447-20130426-07

[25] McConoughey SJ, Howlin RP, Wiseman J *et al.* Comparing PMMA and calcium sulfate as carriers for the local delivery of antibiotics to infected surgical sites. *J Biomed Mater Res B Appl Biomater* 2015; **103**: 870–877. 25142105 10.1002/jbm.b.33247

[26] Nau C, Seebach C, Trumm A *et al.* Alteration of Masquelet’s induced membrane characteristics by different kinds of antibiotic enriched bone cement in a critical size defect model in the rat’s femur. *Injury* 2016; **47**: 325–334. 26652225 10.1016/j.injury.2015.10.079

[27] Rathbone CR, Cross JD, Brown KV *et al.* Effect of various concentrations of antibiotics on osteogenic cell viability and activity. *J Orthop Res* 2011; **29**: 1070–1074. 21567453 10.1002/jor.21343

[28] Aurégan JC, Bégué T. Induced membrane for treatment of critical sized bone defect: a review of experimental and clinical experiences. *Int Orthop* 2014; **38**: 1971–1978. 24984595 10.1007/s00264-014-2422-y

[29] Giannoudis PV, Faour O, Goff T *et al.* Masquelet technique for the treatment of bone defects: tips-tricks and future directions. *Injury* 2011; **42**: 591–598. 21543068 10.1016/j.injury.2011.03.036

[30] Arrington ED, Smith WJ, Chambers HG *et al.* Complications of iliac crest bone graft harvesting. *Clin Orthop Relat Res* 1996; **329**: 300–309. 10.1097/00003086-199608000-00037 8769465

[31] Sen MK, Miclau T. Autologous iliac crest bone graft: should it still be the gold standard for treating nonunions? *Injury* 2007; **38(Suppl 1)**: S75–S80. 17383488 10.1016/j.injury.2007.02.012

[32] Sagi HC, Young ML, Gerstenfeld L *et al.* Qualitative and quantitative differences between bone graft obtained from the medullary canal (with a Reamer/Irrigator/Aspirator) and the iliac crest of the same patient. *J Bone Joint Surg Am* 2012; **94**: 2128–2135. 23224383 10.2106/JBJS.L.00159

[33] Wu H, Whitfield TW, Gordon JA *et al.* Genomic occupancy of Runx2 with global expression profiling identifies a novel dimension to control of osteoblastogenesis. *Genome Biol* 2014; **15**: R52. 24655370 10.1186/gb-2014-15-3-r52 PMC4056528

[34] Toosi S, Naderi-Meshkin H, Kalalinia F *et al.* Comparative characteristics of mesenchymal stem cells derived from reamer-irrigator-aspirator, iliac crest bone marrow, and adipose tissue. *Cell Mol Biol* 2016; **62**: 68–74. 27609477

[35] Le Baron M, Vivona JP, Maman P *et al.* Can the reamer/irrigator/aspirator system replace anterior iliac crest grafting when treating long bone nonunion? *Orthop Traumatol Surg Res* 2019; **105**: 529–533. 30885818 10.1016/j.otsr.2018.12.011

[36] Dawson J, Kiner D, Gardner W *et al.* The Reamer–Irrigator–Aspirator as a device for harvesting bone graft compared with iliac crest bone graft: union rates and complications. *J Orthop Trauma* 2014; **28**: 584–590. 24625833 10.1097/BOT.0000000000000086

[37] Calori GM, Colombo M, Mazza EL *et al.* Incidence of donor site morbidity following harvesting from iliac crest or RIA graft. *Injury* 2014; **45(Suppl 6)**: S116–S120. 25457330 10.1016/j.injury.2014.10.034

[38] Belthur MV, Conway JD, Jindal G *et al.* Bone graft harvest using a new intramedullary system. Clin Orthop Rel Res 2008; **466**: 2973–2980. 10.1007/s11999-008-0538-3 PMC2628246 18841433

[39] Morelli I, Drago L, George DA *et al.* Masquelet technique: myth or reality? A systematic review and meta-analysis. *Injury* 2016; **47(Suppl 6)**: S68–S76. 28040090 10.1016/S0020-1383(16)30842-7

[40] Masquelet AC, Benko PE, Mathevon H *et al.* Harvest of cortico-cancellous intramedullary femoral bone graft using the Reamer-Irrigator-Aspirator (RIA). *Orthop Traumatol Surg Res* 2012; **98**: 227–232. 22402294 10.1016/j.otsr.2012.01.003

[41] Conway JD. Autograft and nonunions: morbidity with intramedullary bone graft versus iliac crest bone graft. *Orthop Clin North Am* 2010; **41**: 75–84. 19931055 10.1016/j.ocl.2009.07.006

[42] Marchand LS, Rothberg DL, Kubiak EN, Higgins TF. Is this autograft worth it?: The blood loss and transfusion rates associated with reamer irrigator aspirator bone graft harvest. *J Orthop Trauma* 2017; **31**: 205–209. 10.1097/BOT.0000000000000811 28166173

[43] Conrad EU, Gretch DR, Obermeyer KR *et al.* Transmission of the hepatitis-C virus by tissue transplantation. *J Bone Joint Surg Am* 1995; **77**: 214–224. 7844127 10.2106/00004623-199502000-00007

[44] Simonds RJ, Holmberg SD, Hurwitz RL *et al.* Transmission of human immunodeficiency virus type 1 from a seronegative organ and tissue donor. *N Engl J Med* 1992; **326**: 726–732. 1738377 10.1056/NEJM199203123261102

[45] Burchardt H. The biology of bone graft repair. *Clin Orthop Relat Res* 1983; **174**: 28–42. 6339139

[46] Giannoudis PV, Dinopoulos H, Tsiridis E. Bone substitutes: an update. *Injury* 2005; **36(Suppl 3)**: S20–S27. 16188545 10.1016/j.injury.2005.07.029

[47] Zimmermann G, Moghaddam A. Allograft bone matrix versus synthetic bone graft substitutes. *Injury* 2011; **42(Suppl 2)**: S16–S21. 21889142 10.1016/j.injury.2011.06.199

[48] Schmidmaier G, Miska M, Zietzschmann S, Moghaddam A. Selection of graft expanders for the second stage of the induced membrane technique. *Tech Orthop* 2016; **31**: 14–22.

[49] Faour O, Dimitriou R, Cousins CA, Giannoudis PV. The use of bone graft substitutes in large cancellous voids: any specific needs? *Injury* 2011; **42(Suppl 2)**: S87–S90. 21723553 10.1016/j.injury.2011.06.020

[50] Zappaterra T, Ghislandi X, Adam A *et al.* Induced membrane technique for the reconstruction of bone defects in upper limb. A prospective single center study of nine cases. *Chir Main* 2011; **30**: 255–263. 21816650 10.1016/j.main.2011.06.005

[51] Cho JW, Kim J, Cho WT *et al.* Circumferential bone grafting around an absorbable gelatin sponge core reduced the amount of grafted bone in the induced membrane technique for critical-size defects of long bones. *Injury* 2017; **48**: 2292–2305. 28802745 10.1016/j.injury.2017.08.012

[52] Mi M, Papakostidis C, Wu X, Giannoudis PV. Mixed results with the Masquelet technique: a fact or a myth? *Injury* 2020; **51**: 132–135. 31883866 10.1016/j.injury.2019.12.032

[53] Accadbled F, Mazeau P, Chotel F *et al.* Induced-membrane femur reconstruction after resection of bone malignancies: three cases of massive graft resorption in children. *Orthop Traumatol Surg Res* 2013; **99**: 479–483. 23608487 10.1016/j.otsr.2013.01.008

[54] Chotel F, Nguiabanda L, Braillon P *et al.* Induced membrane technique for reconstruction after bone tumor resection in children: a preliminary study. *Orthop Traumatol Surg Res* 2012; **98**: 301–308. 22483631 10.1016/j.otsr.2011.11.008

[55] Gouron R, Deroussen F, Plancq MC, Collet LM. Bone defect reconstruction in children using the induced membrane technique: a series of 14 cases. *Orthop Traumatol Surg Res* 2013; **99**: 837–843. 24070692 10.1016/j.otsr.2013.05.005

[56] Mansour TM, Ghanem IB. Preliminary results of the induced membrane technique for the reconstruction of large bone defects. *J Pediatr Orthop* 2017; **37**: e67–e74. 26469687 10.1097/BPO.0000000000000663

[57] Lasanianos NG, Kanakaris NK, Giannoudis PV. Current management of long bone large segmental defects. *Orthop Trauma* 2010; **24**: 149–163.

[58] Durand M, Collombet JM, Mathieu L. Masquelet induced membrane technique for the surgical treatment of large bone defects: the reasons for successes and failures. *Am J Biomed Sci Res* 2019; **2**: 166–169.

[59] Gupta G, Ahmad S, Zahid M *et al.* Management of traumatic tibial diaphyseal bone defect by “induced-membrane technique”. *Indian J Orthop* 2016; **50**: 290–296. 27293290 10.4103/0019-5413.181780 PMC4885298

[60] Assal M, Stern R. The Masquelet procedure gone awry. *Orthopedics* 2014; **37**: e1045–e1048. 25361369 10.3928/01477447-20141023-93

[61] Giannoudis PV, Einhorn TA, Marsh D. Fracture healing: the diamond concept. *Injury* 2007; **38(Suppl 4)**: S3–S6. 10.1016/s0020-1383(08)70003-2 18224731

[62] Harwood PJ, Newman JB, Michael AL. (ii) An update on fracture healing and non-union. *Orthop Trauma* 2010; **24**: 9–23.

[63] Jiang N, Qin CH, Ma YF *et al.* Possibility of one-stage surgery to reconstruct bone defects using the modified Masquelet technique with degradable calcium sulfate as a cement spacer: a case report and hypothesis. *Biomed Rep* 2016; **4**: 374–378. 26998279 10.3892/br.2016.584 PMC4774369

[64] Ren L, Kang Y, Browne C *et al.* Fabrication, vascularization and osteogenic properties of a novel synthetic biomimetic induced membrane for the treatment of large bone defects. *Bone* 2014; **64**: 173–182. 24747351 10.1016/j.bone.2014.04.011 PMC4180017

[65] Aktuglu K, Erol K, Vahabi A. Ilizarov bone transport and treatment of critical-sized tibial bone defects: a narrative review. *J Orthop Traumatol* 2019; **20**: 22. 30993461 10.1186/s10195-019-0527-1 PMC6468024

[66] Einhorn TA. The cell and molecular biology of fracture healing. *Clin Orthop Rel Res* 1998; **355 Suppl**: S7–S21. 10.1097/00003086-199810001-00003 9917622

[67] Einhorn TA, Gerstenfeld LC. Fracture healing: mechanisms and interventions. *Nat Rev Rheumatol* 2015; **11**: 45–54. 25266456 10.1038/nrrheum.2014.164 PMC4464690

[68] Masquelet AC, Fitoussi F, Begue T, Muller GP. Reconstruction of the long bones by the induced membrane and spongy autograft. *Ann Chir Plast Esthet* 2000; **45**: 346–353. 10929461

[69] Judet J, Judet R. The use of an artificial femoral head for arthroplasty of the hip joint. *J Bone Joint Surg Br* 1950; **32-B**: 166–173. 15422013 10.1302/0301-620X.32B2.166

[70] Pinto DA, Vaidya SV, Agashe MV. Preparation of cement spacer in treatment of segmental bone defects in children by the induced membrane technique: a technical note. *J Clin Orthop Trauma* 2021; **15**: 1–8. 33717909 10.1016/j.jcot.2020.12.005 PMC7920131

[71] Gessmann J, Rosteius T, Baecker H *et al.* Is the bioactivity of induced membranes time dependent? *Eur J Trauma Emerg Surg* 2022; **48**: 3051–3061. 34873632 10.1007/s00068-021-01844-4 PMC9360131

[72] Retzepi M, Donos N. Guided Bone Regeneration: biological principle and therapeutic applications. *Clin Oral Implants Res* 2010; **21**: 567–576. 20666785 10.1111/j.1600-0501.2010.01922.x

[73] Gindraux F, Loisel F, Bourgeois M *et al.* Induced membrane maintains its osteogenic properties even when the second stage of Masquelet’s technique is performed later. *Eur J Trauma Emerg Surg* 2020; **46**: 301–312. 31321472 10.1007/s00068-019-01184-4

[74] Powerski M, Maier B, Frank J, Marzi I. Treatment of severe osteitis after elastic intramedullary nailing of a radial bone shaft fracture by using cancellous bone graft in Masquelet technique in a 13-year-old adolescent girl. *J Pediatr Surg* 2009; **44**: E17–E19. 10.1016/j.jpedsurg.2009.04.039 19635286

[75] Daculsi G, Weiss P, Bouler JM *et al.* Biphasic calcium phosphate/hydrosoluble polymer composites: a new concept for bone and dental substitution biomaterials. *Bone* 1999; **25(Suppl 2)**: 59S–61S. 10458277 10.1016/s8756-3282(99)00135-0

[76] Palmieri A, Pezzetti F, Brunelli G *et al.* Calcium sulfate acts on the miRNA of MG63E osteoblast-like cells. *J Biomed Mater Res B Appl Biomater* 2008; **84**: 369–374. 17618507 10.1002/jbm.b.30880

[77] Bouler JM, Pilet P, Gauthier O, Verron E. Biphasic calcium phosphate ceramics for bone reconstruction: a review of biological response. *Acta Biomater* 2017; **53**: 1–12. 28159720 10.1016/j.actbio.2017.01.076

[78] Gerstenfeld LC, Cho TJ, Kon T *et al.* Impaired intramembranous bone formation during bone repair in the absence of tumor necrosis factor-alpha signaling. *Cells Tissues Organs* 2001; **169**: 285–294. 11455125 10.1159/000047893

[79] Lu J, Blary MC, Vavasseur S *et al.* Relationship between bioceramics sintering and micro-particles-induced cellular damages. *J Mater Sci Mater Med* 2004; **15**: 361–365. 15332600 10.1023/b:jmsm.0000021102.68509.65

[80] Samara E, Moriarty TF, Decosterd LA *et al.* Antibiotic stability over six weeks in aqueous solution at body temperature with and without heat treatment that mimics the curing of bone cement. *Bone Joint Res* 2017; **6**: 296–306. 28515059 10.1302/2046-3758.65.BJR-2017-0276.R1 PMC5457644

[81] Cox G, Jones E, McGonagle D, Giannoudis PV. Reamer-irrigator-aspirator indications and clinical results: a systematic review. *Int Orthop* 2011; **35**: 951–956. 21243358 10.1007/s00264-010-1189-z PMC3167404

[82] Finkemeier CG, Neiman R, Hallare D. RIA: one community’s experience. *Orthop Clin North Am* 2010; **41**: 99–103. 19931058 10.1016/j.ocl.2009.07.007

[83] Giannoudis PV, Tzioupis C, Green J. Surgical techniques: how I do it? The Reamer/Irrigator/Aspirator (RIA) system. *Injury* 2009; **40**: 1231–1236. 19783249 10.1016/j.injury.2009.07.070

[84] Qvick LM, Ritter CA, Mutty CE *et al.* Donor site morbidity with reamer-irrigator-aspirator (RIA) use for autogenous bone graft harvesting in a single centre 204 case series. *Injury* 2013; **44**: 1263–1269. 23845569 10.1016/j.injury.2013.06.008

[85] Robison JW, Grau-Cruz EE, Bruggers J, Becher S. Bone graft volume by reamer head size using the RIA 2. *Tech Orthop* 2022; **37**: 149–153.

[86] Verboket RD, Leiblein M, Janko M *et al.* From two stages to one: acceleration of the induced membrane (Masquelet) technique using human acellular dermis for the treatment of non-infectious large bone defects. *Eur J Trauma Emerg Surg* 2020; **46**: 317–327. 31932852 10.1007/s00068-019-01296-x PMC7113234

[87] Fenelon M, Etchebarne M, Siadous R *et al.* Comparison of amniotic membrane versus the induced membrane for bone regeneration in long bone segmental defects using calcium phosphate cement loaded with BMP-2. *Mater Sci Eng C Mater Biol Appl* 2021; **124**: 112032. 33947534 10.1016/j.msec.2021.112032

[88] Gindraux F, Rondot T, de Billy B *et al.* Similarities between induced membrane and amniotic membrane: novelty for bone repair. *Placenta* 2017; **59**: 116–123. 28673520 10.1016/j.placenta.2017.06.340

[89] Ganguly P, Jones E, Panagiotopoulou V *et al.* Electrospun and 3D printed polymeric materials for one-stage critical-size long bone defect regeneration inspired by the Masquelet technique: recent advances. *Injury* 2022; **53(Suppl 2)**: S2–S12. 10.1016/j.injury.2022.02.036 35305805

